# Omicron variant of SARS-COV-2 in Shanghai: Clinical features and inactivated vaccine efficacy in 13,120 elderly patients

**DOI:** 10.7150/ijms.84452

**Published:** 2023-07-24

**Authors:** Jingwen Li, Ru Wen, Guizhu Li, Ying Cao, Zhiqiang Chen, Yaping Chen, Chen Liu

**Affiliations:** 1 Department of Gastroenterology, Southwest Hospital, Army Medical University (Third Military Medical University), Chongqing, 400038, China.; 2 Department of Radiology, Southwest Hospital, Army Medical University (Third Military Medical University), Chongqing, 400038, China.; 3 College of Mathematics and Statistics, Chongqing University, Chongqing, 400044, China.; 4 Department of Intensive Care Medicine, Southwest Hospital, Army Medical University (Third Military Medical University), Chongqing, 400038, China.; 5 Department of Pediatrics, Southwest Hospital, Army Medical University (Third Military Medical University), Chongqing, 400038, China.; 6 Department of Neurosurgery, 958 Hospital, Army Medical University (Third Military Medical University), Chongqing, 400038, China.

**Keywords:** COVID-19, Elderly patients, Inactive vaccine effectiveness, Omicron

## Abstract

**Background:** Few reports concerning inactivated vaccine efficacy in elderly patients with Omicron infection. We aimed at demonstrating the clinical characteristics of elderly patients with mild disease and assessing the protective effect of the vaccine preliminarily.

**Methods:** 13,120 mild patients who aged beyond 60 years old were included in this study totally, medical records were collected and analyzed.

**Results:** Patients beyond 60 years had more chronic comorbidities, significantly lower ORF1ab and N gene CT values, and longer time of nucleic acid conversion than other age groups. Higher CT value of ORF1ab and N gene were found in older patients who received a booster dose of vaccine than in those who received two doses. The time of nucleic acid conversion was longest in unvaccinated old patients, with a decreasing trend from those who received two doses to those who received a booster doses. We also used random forest and logistic regression to screen for factors strongly associated with nucleic acid conversion and to predict the time of nucleic acid conversion.

**Conclusion:** For mild patients with Omicron infection, patients aged>60 years had mild clinical symptoms, higher viral loads, and longer time of nucleic acid conversion, when compared with younger patients. The inactivated SARS-CoV-2 vaccine provided effective protection among adults with omicron variant infection, and the effectiveness of three doses of the vaccine was greater than that of two doses of the vaccine. Special attention should be given to elderly patients.

## Introduction

Age is one of the most important risk factors for severe COVID-19 infection. As of July, 2022, COVID-19 has resulted in more than 6 million deaths worldwide, the average age of deceased patients was around 70 years old 1-3. The elderly people are at a higher risk of COVID-19 infection, with atypical clinical presentation, greater disease severity, and significant mortality 4,5. Broader psychological and physical changes with age, such as a stronger inflammatory response to antigens and a lower ability to inhibit infection^6^,^7^. Aging also promote the expression of angiotensin-converting enzyme 2 (ACE-2) 8, which leads to increase susceptibility to SARS-CoV-2 infection. Moreover, diverse aging-related comorbidities are associated with disease progression and poor prognosis such as cardiovascular disease and diabetes 9,10. Management of the elderly people infected with COVID-19 and related mortality reduction has become a big challenge. At present, mass vaccination programs have been implemented over the world, determining the effectiveness of vaccination in elderly patients has important implications.

Omicron variant was first detected in November 2021 and rapidly spread globally, this variant is associated with a lower risk of hospitalization and death when compared to other variants 11-13. Even so, the prognosis and clinical outcomes of elderly patients with Omicron were worse. Adjei et al reported 81.9% of in-hospital deaths occurred among adults aged ≥65 years and 73.4% occurred among persons with three or more underlying medical conditions during omicron period^14^. Another one larger research showed that the mean age of 7656 deceased cases with omicron variant were over 75-years old in Hong Kong^15^. There are three main types of available vaccines worldwide including mRNA vaccines (Pfizer, Modena), adenovirus vector vaccines (Beijing Institute of Biotechnology, AstraZeneca, Johnson and Johnson), and inactivated vaccines (Sinovac, Sinopharm) 16. With the numerous mutations of Omicron variant, the efficacy of available vaccines has received sustained attention and many studies on this aspect have been conducted. However, few researches concerning the vaccines efficacy for elderly patients were published; meanwhile, the majority of these studies mainly targeted mRNA and adenovirus vector vaccines such as BNT162b2 and mRNA-1273^17^-^19^.

In late March 2022, omicrons wave swept Shanghai first and incurred huge losses. The majority of Chinese people received inactivated vaccine, approximately 85% people ≥60 years have completed primary immunization with 2 does and 67% with a booster vaccine. The effectiveness of inactivated vaccines against Omicron remains unclear, real-world evidence have great significance during omicron wave. In this study, we focus on mild patients aged over 60 years who were admitted to the largest Fangcang shelter hospital in Shanghai, describes the demographics, clinical features and vaccination of these patients, analyses the vaccine efficacy in order to demonstrate the relationship between omicron infection, vaccination and age.

## Methods

### Patient Enrollment

This retrospective study involved mild patients with omicron variant infection, who were admitted to the largest Fangcang shelter hospital from March 28, 2022 to June 28, 2022 in Shanghai. This Fangcang shelter hospital was converted from the Shanghai National Exhibition and Convention Center which is one of the largest Fangcang hospital in China, it was designated to admit patients with mild disease during the outbreak of Omicron. If the condition worsens, the patient will be transferred immediately to another designated hospital for further treatment. All the patients were diagnosed by PCR tests for the ORF1ab and N genes, and the diagnosis was confirmed by positive results for both genes. All admitted patients were discharged only after two consecutive negative PCR tests. The follow-up was terminated at the date of hospital closure. The clinical course classification was according to the Ninth Version of National COVID-19 Guidance. Participants were confirmed to have mild infection when the clinical symptoms were mild and imaging showed no signs of pneumonia. This study adhered to the principles of the Declaration of Helsinki and was approved by the Ethics Committee of Southwest Hospital, Army Medical University (Third Military Medical University) (approval number: KY2022114). The requirement of informed consent was waived for this study by the Ethics Committee of Southwest Hospital, Army Medical University due to the retrospective nature of this study.

### Data collection

Demographic and clinical parameters collected from medical records including demographic information, medical history, comorbidities and clinical symptoms, all data were examined by two independent researchers. PCR test results and cycle threshold (Ct) values were recorded for each SARS-CoV-2 positive sample. The Ct value reflects the number of cycles required to detect viral genetic material during PCR amplification and is inversely proportional to the amount of target nucleic acids in the test sample^20^. We also calculated the mean nucleic acid test Ct values and the time of nucleic acid conversion (the time from the first positive result of the SARS-CoV-2 gene test and the first negative result). The COVID-19 vaccination status (including the number of doses and the brand name of vaccines) was self-reported by patients.

### Statistics

Continuous variables were expressed as mean and standard deviation, while categorical variables were expressed as frequencies and percentages (%). T-test, analysis of variance, and chi-square test were used when appropriate. P-values less than 0.05 were considered statistically significant. Odds ratios and 95% confidence intervals were calculated for each variable. All statistical analyses were performed using *SPSS* Statistics version *20* (*SPSS*, Inc., USA).

### CART regression tree, Logistic regression and Random Forest

Random forest analysis was performed using the random forest package in the R-software with 500 trees and default settings 21. Thirty potential factors that could influence the duration prior to nucleic acid conversion were sequentially included in the random forest model, with the number of trees and random seeds set to 500 and 20, respectively. The final classification results were determined based on the voting scores of each classified tree, and the specificity of the variables and their contribution to the classification of the sample were assessed using the Gini index to achieve importance ranking. Top 15 variables screened by the random forest model were included in the multivariate logistic regression model to screen for variables that were considered closely related to the time of nucleic acid conversion. In assessing the performance of each model, we used the area under the curve (AUC) with a 95% confidence interval (95% CI).

The CART model was used to predict the time of nucleic acid conversion in patients with Omicron infection. The duration prior to nucleic acid conversion was set as the target variable, the other 30 potential predictors were set as predictor variables, and the growth method of CART was selected; second, the data set was divided into a training set and a test set, giving 70% and 30% of the samples, respectively; then, the decision tree was restricted for growth, limiting Maximum Tree depth to 5/4, minimum cases in parent node to 10, and minimum cases in child node to 2; finally, the maximum difference in risk (standard error) was chosen set at 0.2, and the tree was constructed to avoid over-fitting^22^.

## Results

### Demographic and clinical characteristics of adult patients with omicron variant infections

Figure S1 illustrates the chart of this study selection. The demographic and clinical features of patients were summarized (Table S1). A total of 65,439 mild patients with a mean age of 45.8 ± 14.8 years was evaluated. Males accounted for 39,922 (61.0%) and 13,120 (20%) patients were over 60 years old. Of the 13,120 patients who were defined as elderly group, the mean age was 65.7± 4.3years. The elderly group had more comorbidities (p<0.01) and lower frequency clinical symptoms (cough, sputum, fever), when compared with non-elderly groups (p<0.05). The mean Ct values of the ORF1ab and N gene were significantly lower, suggesting a higher viral load in the elderly patients. The mean time of nucleic acid conversion was 6.4 days, which was also significantly longer than that in other age groups.

### Vaccination status of adult patients with omicron variant in different age groups

We next evaluated the vaccination status of all the patients in this study (Table 1). Overall, 50,778 (77.5%) patients were vaccinated and 14,661 (22.5%) patients were unvaccinated. The unvaccinated patients were significantly older (44.2±14.3 vs 49.6±15.8 years, p<0.01) with more comorbidities, demonstrating that the vaccination rates decreased with advancing age. Vaccinated patients showed higher Ct values of both gene and the shorter time of nucleic acid-negative conversion (5.9 vs. 5.3 days). For vaccinated group, patients >60 years presented less clinical signs when compared with the younger group, the opposite trend were shown in the unvaccinated group; and patients >60 years required longer time of nucleic acid conversion in vaccinated and unvaccinated groups. As shown in Table 2, we also evaluated the vaccinated and unvaccinated groups by the age. For patients aged between 18-60 years, vaccinated group presented more clinical signs and shorter time of nucleic acid conversion. The vaccination status of patients aged over 60 years old was assessed. Compared with unvaccinated patients >60 years, vaccinated older patients presented more clinical features, higher CT value of ORF1ab (32.31±2.62 vs 32.04±2.69, p<0.01) and N gene (30.32±2.46 vs 30.05±2.52, p<0.01), and shorter time of nucleic acid conversion (6.1 vs. 6.8 days, p<0.01). Patients >60 years required relatively longer time of nucleic acid conversion whether vaccinated or not, when compared with the younger group.

### Demographic and clinical features of elderly patients in different group based on vaccination status

Totally, 13,120 patients beyond 60 years of age in this study (Table S1), and 8062 (61.4%) patients were vaccinated (Table 1). Three main types of COVID-19 vaccine in China are calculated in this study, including inactivated vaccines (Sinopharm, Sinovac), recombinant protein subunit (Zhifei Longcom) and adenovirus-vectored vaccines (CanSino). Among these elderly patients ,7979 patients received inactivated vaccines (226 patients received only one dose and failed to complete primary vaccination), 28 patients received recombinant protein vaccines, and 55 patients received adenovirus-vector vaccines.

**Figure 1:**
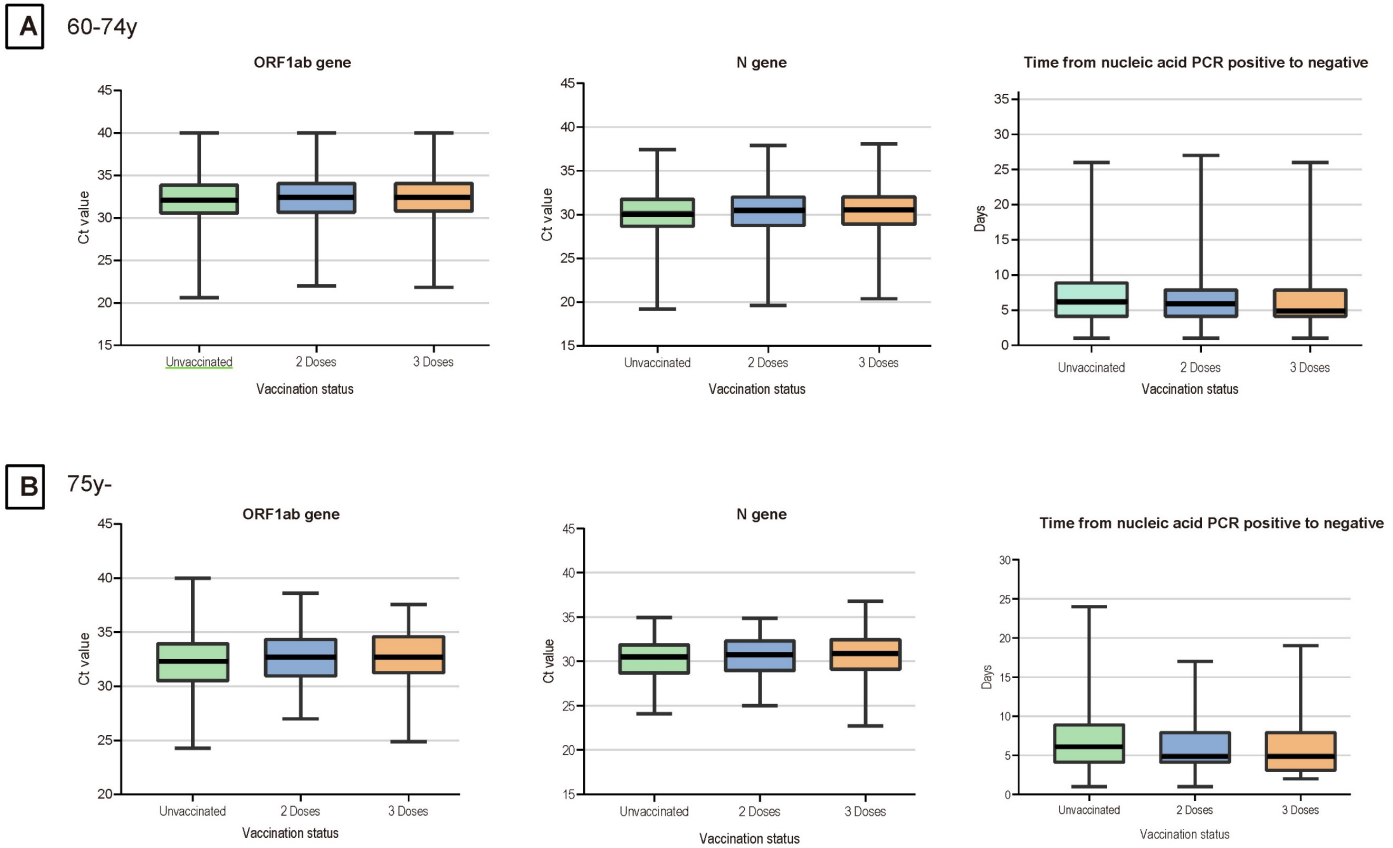
** Ct Values for the N Genes, ORF1ab Genes and the Time of Nucleic Acid Conversion in Elderly Patients.** For each plot, from top to bottom, lines in the box represent the 75th percentile, median, and 25th percentile. The whiskers extend to the largest and smallest values up to 1.5 times the interquartile range from the 75th and 25th percentiles, respectively. Cycle threshold (Ct) values were compared between 3 doses and unvaccinated, 3 doses and 2 doses, and 2 doses and unvaccinated.

Given the small number of patients with the recombinant protein vaccines and adenovirus vaccines, we considered patients who received inactivated vaccination as our main study population, including patients who received two primary doses and a booster dose (patients who failed to complete primary vaccination were excluded). As was shown in Table 3, a total of 3322 (25.9%) patients received two doses of vaccination, 4431 (34.6%) patients received booster dose. For the 60-74 years age group, patients with a booster dose of vaccine exhibited more clinical presentations, higher Ct values of ORF1ab and N gene, and less time of nucleic acid conversion. Patients who aged over 74 years age group also showed a similar trend. Notably, the time of nucleic acid conversion was longest in the unvaccinated group, shortest in the three-dose booster patients, and intermediate in the two-dose patients (p < 0.01) (Figure 1).

### Factors Associated with the time of nucleic acid conversion

We initially screening for important factors associated with the time of nucleic acid conversion in patients beyond 60 years using random forest analysis, the result was showed in Figure S2. Then the top 15 variables of the ranked in random forest were included in multivariate logistic regression model. N gene, respiratory disease and vaccination (p<0.05) were strongly associated with the time of nucleic acid conversion (Table S2). The area under the ROC curve of the regression model was 73.1%, indicating that the logistic regression model had a good predictive accuracy.

### Prediction of the time of nucleic acid conversion

**Figure 2:**
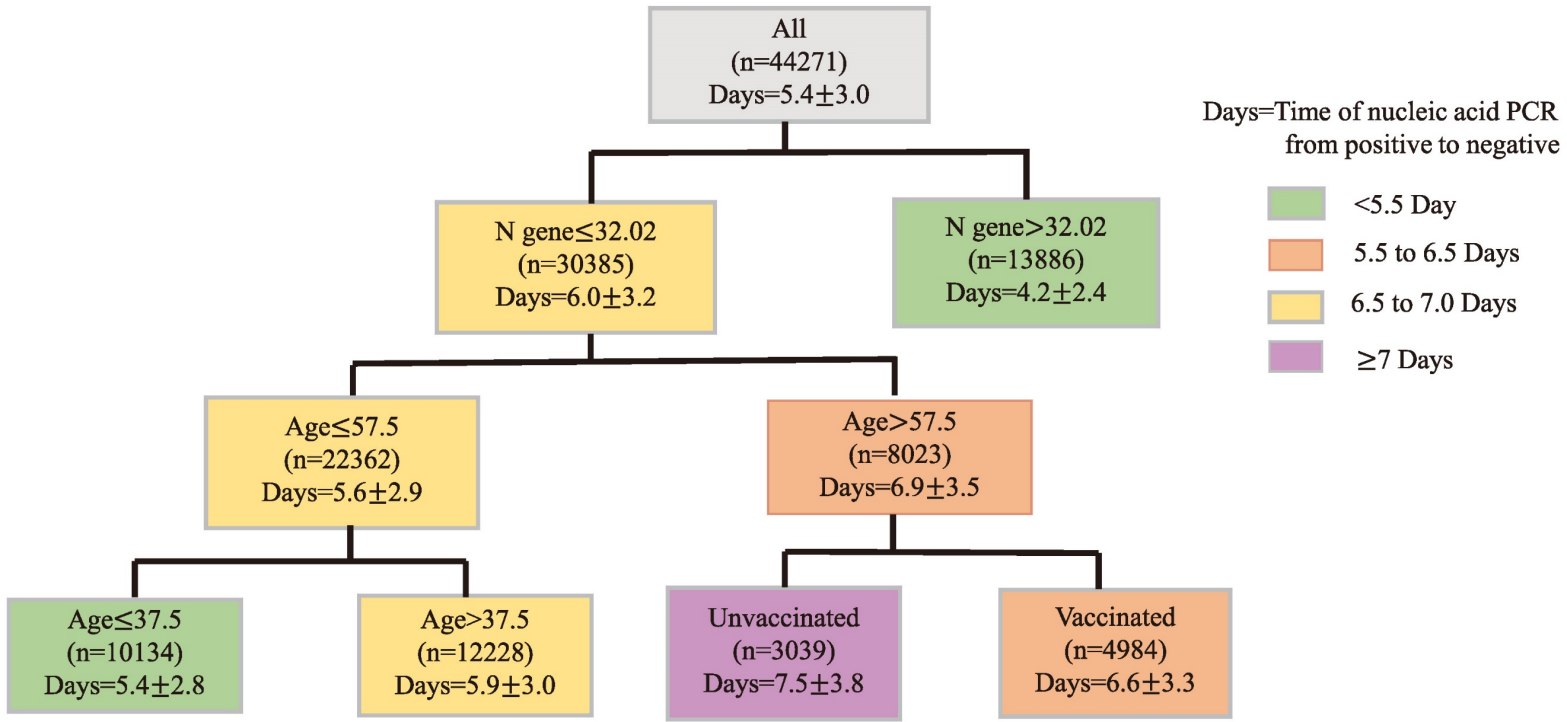
** Prediction of the Time of Nucleic Acid Conversion using CART Analysis.** Regression tree (CART)-derived predictors of time of nucleic acid PCR from positive to negative (Days) in all Omicron patients. Each branch shows the classification variable and each node shows the number of subjects and the estimated days. Colors are used for descriptive purposes only.

Finally, CART regression tree was used to predict the time required for nucleic acid conversion for all patients in this study. As shown in the Figure 2, the decision tree results presented the main predictors influencing the time of nucleic acid conversion. The three factors including N gene, age, and vaccination appeared on the pivot point classification. An N gene Ct value equal to 32.02, age of 57.5 years and 37.5 years were identified as the main predictors of nucleic acid conversion. The shortest time of nucleic acid regression was 4.2 days for N gene Ct values beyond 34.02; and the longest was 7.5 days for unvaccinated patients older than 57.5 years old.

## Discussion

Our results show that among mild patients with omicron variant infection, older patients have more comorbidities, higher viral load, and longer time of nucleic acid conversion. Meanwhile, the effects of omicron variant on the various body systems are relatively less, the clinical symptoms are mild and mainly focus on the respiratory system. Compared to elderly patients who received a booster dose of vaccine, patients who received two doses had lower Ct values of ORF1ab and N genes, and longer time of nucleic acid regression. The inactivated vaccine provided effective protection for omicron-infected elderly patients, and a booster dose after primary course increased the protection. To our best knowledge, this is the first real-world study to investigate inactivated vaccines in elderly people with Omicron infection and characterize the risk factors for the time of the nucleic acid conversion.

There are relatively few reports about the vaccine efficacy of COVID-19 vaccines in elderly patients with Omicron infection. One large-scale research suggested two doses of ChAdOx1 nCoV-19 or BNT162b2 vaccine provided limited protection against omicron variant, a booster increase the protection and the effectiveness protection waned over time^23^. Another report suggested that effectiveness of full vaccination was 96% for Pfizer-BioNTech, 96% for Moderna, and 84% for Janssen vaccine products respectively^24^.

Many of these studies were case-controlled designs, and excluded older people with morbidities and frailty, leaving insufficient published data on safety and efficacy in this population. Our study showed that the inactivated vaccine could reduce the viral loads and shorten the time of nucleic acid conversion for patients aged over 60 years old, the effectiveness of a booster vaccine was superior to those of primary vaccine. Those evidences demonstrated inactivated vaccine is effective for elderly patients during omicron epidemic and a booster vaccine increased protection in China.

More importantly, our results suggested that vaccination rates among adults >60 years of age and above are significantly lower than those in younger age groups. When compared with the younger group, elderly patients needed longer time of nucleic acid conversion whether receiving vaccination or not. Qin et al. indicated that 17.2% of people aged 60 years and above in China were hesitant to receive a booster dose of COVID-19 vaccine, strongly associated with lower levels of perceived susceptibility and benefit and higher levels of perceived impairment. Concerns about contraindications, vaccine safety, and exercise limitations are the main reasons for vaccine hesitation among older Chinese people 25 .Similar to previous studies, this study showed that the clinical symptoms of omicron in elderly patients was less frequent and more insidious compared to younger patients, elderly people with asymptomatic infections becoming an important source of virus transmission 26,27. Given older age was associated with more combabilities, and reduced immunity and greater infection-fatality risk, it is critical to improve the vaccination rates among older people by raising awareness about the sensitivity, efficacy, and safety of the vaccine itself through various communication and education methods (e.g., social media and offline lectures).

This study had some limitations. Firstly, the single-center retrospective study only assessed mild patients at Fangcang shelter hospital in Shanghai, data on patients with moderate to severe Omicron infection were missing; Secondly data about blood biochemical tests and imaging were not available, making the assessment of the severity of Omicron disease progression inadequate. Thirdly, the present study was a cross-sectional study.

## Conclusions

In conclusion, elderly patients had more comorbidities, mild clinical symptoms, higher viral loads, and longer time of nucleic acid conversion. The inactivated vaccination was protective against mild SARS-CoV-2 omicron variant infection in adults beyond 60 years of age. Our findings support the maximization of the booster vaccine coverage in elderly people.

## Supplementary Material

Supplementary figures and tables.

## Figures and Tables

**Table 1:** Demographic and clinical features of covid-19 patients >18 years old according to vaccination status.

Characteristic	Total			P value	Vaccinated		P value	Unvaccinated		P value
		vaccinated	unvaccinated		18-60y	>60y		18-60y	>60y	
	n=65439	n=50778	n=14661		n=42716	n=8062		n=9603	n=5058	
Age, median years (IQR)	45.5(14.8)	44.2(14.3)	49.6(15.8)	<0.01	40.4(11.8)	65.9(3.8)	<0.01	41.0(11.9)	66.8(4.6)	<0.01
Sex										
Female	25517(38.9)	19528(38.5)	5989(40.8)	<0.01	15926(37.3)	3602(44.7)	<0.01	3627(37.8)	2362(46.7)	<0.01
Male	39922(61.0)	31250(61.5)	8672(59.2)		26790(62.7)	4460(55.3)		5976(62.2)	2696(53.3)	
Comorbidity										
Hypertension	7043(10.8)	5124(10.1)	1919(13.1)	<0.01	2720(6.4)	2404(29.8)	<0.01	527(5.5)	1392(27.5)	<0.01
Diabetes	2475(3.8)	1611(3.2)	864(5.9)	<0.01	807(1.9)	804(10)	<0.01	249(2.6)	615(12.2)	<0.01
Heart condition	3042(4.7)	2080(4.1)	962(6.6)	<0.01	1308(3.1)	772(9.6)	<0.01	312(3.2)	650(12.9)	<0.01
Respiratory disease	461(0.7)	299(0.6)	162(1.1)	<0.01	176(0.4)	123(1.5)	<0.01	60(0.6)	102(2.0)	0.08
Hypothyroidism	101(0.1)	60(0.1)	41(0.3)	<0.01	46(0.1)	14(0.2)	0.11	28(0.3)	13(0.3)	<0.01
Renal diseases	152(0.2)	84(0.2)	68(0.5)	<0.01	56(0.1)	28(0.3)	<0.01	33(0.3)	35(0.7)	<0.01
Oncology	149(0.2)	55(0.1)	94(0.6)	<0.01	32(0.1)	23(0.3)	<0.01	33(0.3)	61(1.2)	<0.01
Cerebrovascular disease disease	518(0.8)	359(0.7)	159(1.1)	<0.01	244(0.6)	115(1.4)	<0.01	68(0.7)	91(1.8)	<0.01
Surgery	138(0.2)	89(0.2)	49(0.3)	<0.01	60(0.1)	29(0.4)	<0.01	22(0.2)	27(0.5)	<0.01
Allergies	2396(3.7)	1800(3.5)	596(4.1)	<0.05	1414(3.3)	386(4.8)	<0.01	269(2.8)	327(6.5)	<0.01
Clinical Features										
Cough	12450(19.0)	10983(21.6)	1467(10.0)	<0.01	9721(22.8)	1262(15.7)	<0.01	883(9.2)	584(11.5)	<0.01
Sputum	8125(12.4)	7159(14.1)	966(6.6)	<0.01	6408(15)	751(9.3)	<0.01	584(6.1)	382(7.6)	<0.01
Fatigue	4723(7.2)	4062(8.0)	661(4.5)	<0.01	3694(8.6)	368(4.6)	<0.01	454(4.7)	207(4.1)	0.08
Fever	4104(6.3)	3507(6.9)	597(4.1)	<0.01	3069(7.2)	438(5.4)	<0.01	358(3.7)	239(4.7)	<0.01
Myalgia	3860(5.9)	3351(6.6)	509(3.5)	<0.01	3068(7.2)	283(3.5)	<0.01	362(3.8)	147(2.9)	<0.01
Sore throat	2265(3.5)	2014(4)	251(1.7)	<0.01	1863(4.4)	151(1.9)	<0.01	187(1.9)	64(1.3)	<0.01
Gastrointestinal symptoms	102(0.2)	88(0.2)	14(0.1)	<0.05	83(0.2)	5(0.1)	<0.01	9(0.1)	5(0.1)	0.16
Dyspnea	41(0.1)	35(0.1)	6(0)	0.23	34(0.1)	1(0.0)	0.03	5(0.1)	1(0)	0.67
Chest tightness	67(0.1)	59(0.1)	8(0.1)	<0.05	49(0.1)	10(0.1)	0.86	7(0.1)	1(0)	0.28
Hyposmia	217(0.3)	193(0.4)	24(0.2)	0.22	185(0.4)	8(0.1)	<0.01	20(0.2)	4(0.1)	0.08
Taste perversion	228(0.3)	203(0.4)	25(0.2)	<0.01	192(0.4)	11(0.1)	<0.01	20(0.2)	5(0.1)	0.15
Omicron variant ORF1ab gene cycle threshold (ct Value)	32.60(2.68)	32.68(2.67)	32.36(2.71)	<0.01	32.75(2.67)	32.31(2.62)	<0.01	32.52(2.70)	32.04(2.69)	<0.01
Omicron variant N gene cycle threshold (ct Value)	30.61(2.49)	30.69(2.48)	30.36(2.51)	<0.01	30.77(2.48)	30.32(2.46)	<0.01	30.52(2.50)	30.05(2.52)	<0.01
Time from nucleic acid PCR positive to negative (Days)	5.4 (3.0)	5.3(2.9)	5.9 (3.3)	<0.01	5.1(2.8)	6.1(3.3)	<0.01	5.4(3.0)	6.8(3.8)	<0.01

**Table 2:** Demographic and clinical features of covid-19 patients >18 years old according to age group.

Characteristic	Total			P value	18-60y		P value	>60y		P value
		vaccinated	unvaccinated		vaccinated	unvaccinated		vaccinated	unvaccinated	
	n=65439	n=50778	n=14661		n=42716	n=9603		n=8062	n=5058	
Age, median years (IQR)	45.5(14.8)	44.2(14.3)	49.6(15.8)	<0.01	40.4(11.8)	41.0(11.9)	<0.01	65.9(3.8)	66.8(4.6)	<0.01
Sex										
Female	25517(38.9)	19528(38.5)	5989(40.8)	<0.01	15926(37.3)	3627(37.8)	0.38	3602(44.7)	2362(46.7)	<0.05
Male	39922(61.0)	31250(61.5)	8672(59.2)		26790(62.7)	5976(62.2)		4460(55.3)	2696(53.3)	
Comorbidity										
Hypertension	7043(10.8)	5124(10.1)	1919(13.1)	<0.01	2720(6.4)	527(5.5)	<0.05	2404(29.8)	1392(27.5)	<0.05
Diabetes	2475(3.8)	1611(3.2)	864(5.9)	<0.01	807(1.9)	249(2.6)	<0.01	804(10)	615(12.2)	<0.01
Heart condition	3042(4.7)	2080(4.1)	962(6.6)	<0.01	1308(3.1)	312(3.2)	<0.01	772(9.6)	650(12.9)	<0.05
Respiratory disease	461(0.7)	299(0.6)	162(1.1)	<0.01	176(0.4)	60(0.6)	<0.05	123(1.5)	102(2.0)	<0.05
Hypothyroidism	101(0.1)	60(0.1)	41(0.3)	<0.01	46(0.1)	28(0.3)	<0.01	14(0.2)	13(0.3)	0.31
Renal diseases	152(0.2)	84(0.2)	68(0.5)	<0.01	56(0.1)	33(0.3)	<0.01	28(0.3)	35(0.7)	<0.05
Oncology	149(0.2)	55(0.1)	94(0.6)	<0.01	32(0.1)	33(0.3)	<0.01	23(0.3)	61(1.2)	<0.01
Cerebrovascular disease	518(0.8)	359(0.7)	159(1.1)	<0.01	244(0.6)	68(0.7)	0.12	115(1.4)	91(1.8)	0.10
Surgery	138(0.2)	89(0.2)	49(0.3)	<0.01	60(0.1)	22(0.2)	<0.05	29(0.4)	27(0.5)	0.14
Allergies	2396(3.7)	1800(3.5)	596(4.1)	<0.05	1414(3.3)	269(2.8)	<0.05	386(4.8)	327(6.5)	<0.01
Clinical Features										
Cough	12450(19.0)	10983(21.6)	1467(10.0)	<0.01	9721(22.8)	883(9.2)	<0.01	1262(15.7)	584(11.5)	<0.01
Sputum	8125(12.4)	7159(14.1)	966(6.6)	<0.01	6408(15)	584(6.1)	<0.01	751(9.3)	382(7.6)	<0.01
Fatigue	4723(7.2)	4062(8.0)	661(4.5)	<0.01	3694(8.6)	454(4.7)	<0.01	368(4.6)	207(4.1)	0.20
Fever	4104(6.3)	3507(6.9)	597(4.1)	<0.01	3069(7.2)	358(3.7)	<0.01	438(5.4)	239(4.7)	0.08
Myalgia	3860(5.9)	3351(6.6)	509(3.5)	<0.01	3068(7.2)	362(3.8)	<0.01	283(3.5)	147(2.9)	0.06
Sore throat	2265(3.5)	2014(4)	251(1.7)	<0.01	1863(4.4)	187(1.9)	<0.01	151(1.9)	64(1.3)	<0.05
Gastrointestinal symptoms	102(0.2)	88(0.2)	14(0.1)	<0.05	83(0.2)	9(0.1)	0.15	5(0.1)	5(0.1)	0.59
Dyspnea	41(0.1)	35(0.1)	6(0)	0.23	34(0.1)	5(0.1)	0.37	1(0.0)	1(0)	0.74
Chest tightness	67(0.1)	59(0.1)	8(0.1)	<0.05	49(0.1)	7(0.1)	0.26	10(0.1)	1(0)	<0.05
Hyposmia	217(0.3)	193(0.4)	24(0.2)	0.22	185(0.4)	20(0.2)	<0.05	8(0.1)	4(0.1)	0.71
Taste perversion	228(0.3)	203(0.4)	25(0.2)	<0.01	192(0.4)	20(0.2)	<0.05	11(0.1)	5(0.1)	0.55
Omicron variant ORF1ab gene cycle threshold (ct Value)	32.60(2.68)	32.68(2.67)	32.36(2.71)	<0.01	32.75(2.67)	32.52(2.70)	<0.01	32.31(2.62)	32.04(2.69)	<0.01
Omicron variant N gene cycle threshold (ct Value)	30.61(2.49)	30.69(2.48)	30.36(2.51)	<0.01	30.77(2.48)	30.52(2.50)	<0.01	30.32(2.46)	30.05(2.52)	<0.01
Time from nucleic acid PCR positive to negative (Days)	5.4 (3.0)	5.3(2.9)	5.9 (3.3)	<0.01	5.1(2.8)	5.4(3.0)	<0.01	6.1(3.3)	6.8(3.8)	<0.01

**Table 3:** Demographic and clinical features of elderly patients with covid-19 in different groups according to vaccination status.

Characteristic	Overall (>60y) (n=12811)	Vaccinated				Unvaccinated		P value
		2 doses		3 doses		0 dose		
		60-74y	>74y	60-74y	>74y	60-74y	>74y	
		(n=3233)	(n=89)	(n=4325)	(n=106)	(n=4773)	(n=285)	
Age (IQR)	66.3(4.1)	65.6(3.2)	77.2(3.0)	65.6(3.1)	77.3(2.9)	66.0(3.3)	78.6(3.6)	<0.01
Sex								
Female	5829(45.5)	1535(47.5)	35(39.3)	1857(42.9)	40(37.7)	2219(46.5)	143(50.2)	<0.01
Male	6982(54.5)	1698(52.5)	54(60.7)	2468(57.1)	66(62.3)	2554(53.5)	142(49.8)	
Comorbidity								
Hypertension	3717(29.0)	1018(31.5)	43(48.3)	1213(28.0)	51(48.1)	1270(26.6)	122(42.8)	<0.01
Diabetes	1390(10.9)	334(10.3)	16(18)	412(9.5)	13(12.3)	562(11.8)	53(18.6)	<0.01
Heart condition	1394(10.9)	324(10.0)	9(10.1)	387(8.9)	24(22.6)	581(12.2)	69(24.2)	<0.01
Respiratory disease	221(1.7)	48(1.5)	3(3.4)	66(1.5)	2(1.9)	97(2.0)	5(1.8)	0.29
Hypothyroidism	27(0.2)	7(0.2)	0(0.0)	6(0.1)	1(0.9)	13(0.3)	0(0.0)	0.37
Renal diseases	61(0.5)	17(0.5)	0(0.0)	8(0.2)	1(0.9)	32(0.7)	3(1.1)	0.01
Oncology	84(0.7)	11(0.3)	1(1.1)	11(0.3)	0(0.0)	55(1.2)	6(2.1)	<0.01
Cerebrovascular disease	199(1.6)	45(1.4)	1(1.1)	59(1.4)	3(2.8)	80(1.7)	11(3.9)	<0.01
Surgery	55(0.4)	12(0.4	0(0.0)	16(0.4)	0(0.0)	27(0.6)	0(0.0)	0.44
Allergies	701(5.5)	168(5.2)	6(6.7)	197(4.6)	3(2.8)	292(6.1)	35(12.3)	<0.01
Clinical Features								
Cough	1799(14)	477(14.8)	20(22.5)	689(15.9)	29(27.4)	533(11.2)	51(17.9)	<0.01
Sputum	1108(8.6)	289(8.9)	12(13.5)	404(9.3)	21(19.8)	343(7.2)	39(13.7)	<0.01
Fatigue	560(4.4)	156(4.8)	8(9)	182(4.2)	7(6.6)	189(4)	18(6.3)	0.03
Fever	656(5.1)	188(5.8)	4(4.5)	214(4.9)	11(10.4)	230(4.8)	9(3.2)	0.03
Myalgia	420(3.3)	110(3.4)	6(6.7)	152(3.5)	5(4.7)	140(2.9)	7(2.5)	0.20
Sore throat	210(1.6)	53(1.6)	0(0.0)	91(2.1)	2(1.9)	59(1.2)	5(1.8)	0.03
Gastrointestinal symptoms	7(0.1)	5(0.2)	0(0.0)	0(0.0)	0(0.0)	2(0.0)	0(0,0)	0.12
Dyspnea	2(0.0)	0(0.0)	0(0.0)	1(0.0)	0(0.0)	1(0.0)	0(0.0)	0.98
Chest tightness	10(0.1)	5(0.2)	0(0.0)	4(0.1)	0(0.0)	1(0.0)	0(0.0)	0.43
Hyposmia	12(0.1)	2(0.1)	0(0.0)	6(0.1)	0(0.0)	4(0.1)	0(0.0)	0.88
Taste perversion	12(0.1)	2(0.1)	0(0.0)	9(0.2)	0(0.0)	0(0.0)	1(0.4)	0.36
Omicron variant ORF1ab gene cycle threshold (ct Value)	32.18(2.66)	32.25(2.71)	32.44(2.57)	32.29(2.57)	32.66(2.58)	32.04(2.69)	31.97(2.72)	<0.01
Omicron variant N gene cycle threshold (ct Value)	30.20(2.50)	30.25(2.54)	30.44(2.41)	30.32(2.43)	30.75(2.53)	30.06(2.52)	30.01(2.53)	<0.01
Time of nucleic acid PCR from positive to negative (Days)	6.4(3.5)	6.1(3.4)	6.5 (4.1)	6.0(3.2)	6.0(3.4)	6.8(3.7)	7.0(4.2)	<0.01

## References

[2] O'Driscoll M, Ribeiro Dos Santos G, Wang L (2021). Age-specific mortality and immunity patterns of SARS-CoV-2. Nature.

[3] Goodman KE, Magder LS, Baghdadi JD (2021). Impact of Sex and Metabolic Comorbidities on Coronavirus Disease 2019 (COVID-19) Mortality Risk Across Age Groups: 66 646 Inpatients Across 613 US Hospitals. Clinical Infectious Diseases.

[4] Grasselli G, Greco M, Zanella A (2020). Risk Factors Associated With Mortality Among Patients With COVID-19 in Intensive Care Units in Lombardy, Italy. JAMA Internal Medicine.

[5] Zhou F, Yu T, Du R (2020). Clinical course and risk factors for mortality of adult inpatients with COVID-19 in Wuhan, China: a retrospective cohort study. The Lancet.

[6] Nikolich-Žugich J (2018). The twilight of immunity: emerging concepts in aging of the immune system. Nature Immunology.

[7] Borgoni S, Kudryashova KS, Burka K, de Magalhães JP (2021). Targeting immune dysfunction in aging. Ageing Research Reviews.

[8] Farshbafnadi M, Kamali Zonouzi S, Sabahi M, Dolatshahi M, Aarabi MH (2021). Aging & COVID-19 susceptibility, disease severity, and clinical outcomes: The role of entangled risk factors. Experimental Gerontology.

[9] Niu S, Tian S, Lou J (2020). Clinical characteristics of older patients infected with COVID-19: A descriptive study. Archives of Gerontology and Geriatrics.

[10] Jain V, Yuan J-M (2020). Predictive symptoms and comorbidities for severe COVID-19 and intensive care unit admission: a systematic review and meta-analysis. International Journal of Public Health.

[11] Burki TK (2022). Omicron variant and booster COVID-19 vaccines. The Lancet Respiratory Medicine.

[12] Nyberg T, Ferguson NM, Nash SG (2022). Comparative analysis of the risks of hospitalisation and death associated with SARS-CoV-2 omicron (B. 1.1. 529) and delta (B. 1.617. 2) variants in England: a cohort study. The Lancet.

[13] Lauring AS, Tenforde MW, Chappell JD (2022). Clinical severity of, and effectiveness of mRNA vaccines against, covid-19 from omicron, delta, and alpha SARS-CoV-2 variants in the United States: prospective observational study. bmj.

[14] Adjei S HK, Molinari NM (2022). Mortality Risk Among Patients Hospitalized Primarily for COVID-19 During the Omicron and Delta Variant Pandemic Periods — United States, April 2020-June 2022. MMWR Morb Mortal Wkly Rep.

[15] Mesfin Y, Chen D, Bond H (2022). Epidemiology of infections with SARS-CoV-2 Omicron BA. 2 variant in Hong Kong, January-March 2022. medRxiv.

[16] Fiolet T, Kherabi Y, MacDonald C-J, Ghosn J, Peiffer-Smadja N (2021). Comparing COVID-19 vaccines for their characteristics, efficacy and effectiveness against SARS-CoV-2 and variants of concern: A narrative review. Clinical Microbiology and Infection.

[17] Sharif N, Alzahrani KJ, Ahmed SN, Dey SK Efficacy, immunogenicity and safety of COVID-19 vaccines: a systematic review and meta-analysis. Frontiers in Immunology. 2021:4149.

[18] Tregoning JS, Flight KE, Higham SL, Wang Z, Pierce BF (2021). Progress of the COVID-19 vaccine effort: viruses, vaccines and variants versus efficacy, effectiveness and escape. Nature Reviews Immunology.

[19] Feikin D, Higdon MM, Abu-Raddad LJ Duration of effectiveness of vaccines against SARS-CoV-2 infection and COVID-19 disease: results of a systematic review and meta-regression. 2021.

[20] Accorsi EK, Britton A, Fleming-Dutra KE (2022). Association Between 3 Doses of mRNA COVID-19 Vaccine and Symptomatic Infection Caused by the SARS-CoV-2 Omicron and Delta Variants. JAMA.

[21] Liaw A, Wiener M (2002). Classification and regression by randomForest. R news.

[22] Swaminathan S, Pasipanodya JG, Ramachandran G (2016). Drug concentration thresholds predictive of therapy failure and death in children with tuberculosis: bread crumb trails in random forests. Clinical Infectious Diseases.

[23] Andrews N, Stowe J, Kirsebom F (2022). Covid-19 vaccine effectiveness against the Omicron (B. 1.1. 529) variant. New England Journal of Medicine.

[24] Moline HL, Whitaker M, Deng L (2021). Effectiveness of COVID-19 vaccines in preventing hospitalization among adults aged≥ 65 years—COVID-NET, 13 states, February-April 2021. Morbidity and Mortality Weekly Report.

[25] Qin C, Yan W, Tao L, Liu M, Liu J (2022). The Association between Risk Perception and Hesitancy toward the Booster Dose of COVID-19 Vaccine among People Aged 60 Years and Older in China. Vaccines.

[26] Mori H, Obinata H, Murakami W (2021). Comparison of COVID-19 disease between young and elderly patients: Hidden viral shedding of COVID-19. Journal of infection and chemotherapy: official journal of the Japan Society of Chemotherapy.

[27] Hwang J, Ryu HS, Kim HA, Hyun M, Lee JY, Yi HA (2020). Prognostic Factors of COVID-19 Infection in Elderly Patients: A Multicenter Study. Journal of clinical medicine.

