# Supplementary material for: Omicron variant of SARS-COV-2 in Shanghai: Clinical features and inactivated vaccine efficacy in 13,120 elderly patients
Source: Int J Med Sci. 2023 Jul 24;20(9):1144–51. doi: 10.7150/ijms.84452 (PMC10416717; doi:10.7150/ijms.84452)
Supplement: Supplementary file 1 — Supplementary figures and tables. [file ijmsv20p1144s1.pdf]

Table S1. Demographic and clinical features of patients with Coivd-19 in different age groups.

Characteristic	Total (n=65439)	18-30y (n=13512)	30-45y (n=17846)	45-60y (n=20961)	>60y (n=13120)	P value
**Age, median years (IQR)**	45.5(14.8)	24.4(3.2)	35.9(3.9)	51.6(3.8)	65.7(4.3)	<0.01
**Sex**						
**Female**	25517(38.9)	4282(31.7)	6708(37.6)	8563(40.9)	5964(45.5)	
**Male**	39922(61.0)	9230(68.3)	11138(62.4)	12398(59.1)	7156(54.5)	
**Comorbidity**						
**Hypertension**	7043(10.8)	82(0.6)	622(3.5)	2543(12.1)	3796(28.9)	<0.01
**Diabetes**	2475(3.8)	25(0.2)	231(1.3)	800(3.8)	1419(10.8)	<0.01
**Heart condition**	3042(4.7)	199(1.5)	495(2.8)	926(4.42)	1422(10.8)	<0.01
**Respiratory disease**	461(0.7)	42(0.3)	70(0.4)	124(0.6)	225(1.7)	<0.01
**Hypothyroidism**	101(0.1)	14(0.1)	31(0.2)	29(0.1)	27(0.2)	0.14
**Renal diseases**	152(0.2)	12(0.1)	39(0.2)	38(0.2)	63(0.5)	0.02
**Oncology**	149(0.2)	4(0.0)	15(0.1)	46(0.2)	84(0.6)	<0.01
**Cerebrovascular disease**	518(0.8)	28(0.2)	101(0.6)	183(0.9)	206(1.6)	<0.01
**Surgery**	138(0.2)	11(0.1)	28(0.2)	43(0.2)	56(0.4)	<0.01
**Allergies**	2396(3.7)	423(3.1)	637(3.6)	623(3.0)	713(5.4)	<0.01
**Clinical Features**						
**Cough**	12450(19.0)	3442(25.5)	3887(21.8)	3275(15.6)	1846(14.1)	<0.01
**Sputum**	8125(12.4)	2196(16.3)	2585(14.5)	2211(10.5)	1133(8.6)	<0.01

**Fatigue**	4723(7.2)	1276(9.4)	1690(9.5)	1182(5.6)	575(4.4)	<0.01
**Fever**	4104(6.3)	1030(7.6)	1264(7.1)	1133(5.4)	677(5.2)	<0.01
**Myalgia**	3860(5.9)	933(6.9)	1367(7.7)	1130(5.4)	430(3.3)	<0.01
**Sore throat**	2265(3.5)	812(6.0)	802(4.5)	436(2.1)	215(1.6)	<0.01
**Gastrointestinal symptoms**	102(0.2)	32(0.2)	31(0.2)	32(0.2)	7(0.1)	<0.01
**Dyspnea**	41(0.1)	17(0.1)	17(0.1)	5(0.0)	2(0.0)	<0.01
**Chest tightness**	67(0.1)	11(0.1)	29(0.2)	16(0.1)	11(0.1)	0.05
**Nausea**	36(0.1)	10(0.1)	14(0.1)	10(0.0)	2(0.0)	0.08
**Hyposmia**	217(0.3)	67(0.5)	97(0.5)	41(0.2)	12(0.1)	<0.01
**Taste perversion**	228(0.3)	67(0.5)	101(0.6)	44(0.2)	16(0.1)	<0.01
**Omicron variant ORF1ab gene cycle threshold (ct Value)**	32.61(2.68)	32.80(2.76)	32.73(2.70)	32.65(2.61)	32.20(2.65)	<0.01
**Omicron variant N gene cycle threshold (ct Value)**	30.61(2.49)	30.82(2.55)	30.74(2.50)	30.65(2.42)	30.21(2.49)	<0.01
**Time of nucleic acid PCR from positive to negative（Days）**	5.4 (3.0)	4.8 (2.7)	5.0 (2.8)	5.5 (3.0)	6.4 (3.5)	<0.01

Data are n (%) and mean (SD).

Table S2. Multinomial logistic regression on factors associated with the time of nucleic acid PCR from positive to negative.

	OR (95% CI)	P value
**Cough**		
**No**	1.00	
**Yes**	0.814(0.719~0.92)	0.001
**Myalgia**		
**No**	1.00	
**Yes**	1.505(1.206~1.876)	<0.001
**Diabetes**		
**No**	1.00	
**Yes**	1.241(1.095~1.407)	<0.001
**Respiratory disease**		
**No**	1.00	
**Yes**	1.425(1.071~1.887)	0.014
**Sex**		
**Female**	1.00	
**Male**	1.211(1.124~1.304)	<0.001
**Age**	1.013(1.005~1.022)	0.016
**Vaccination**		
**0 dose**	1.00	
**2 doses**	0.714 (0.647~0.786)	<0.001
**3 doses**	0.626 (0.572~0.685)	<0.001
**N Gene**	0.745 (0.699~0.793)	<0.001
**ORF1ab gene**	1.093 (1.031~1.159)	0.003

Figure S1.

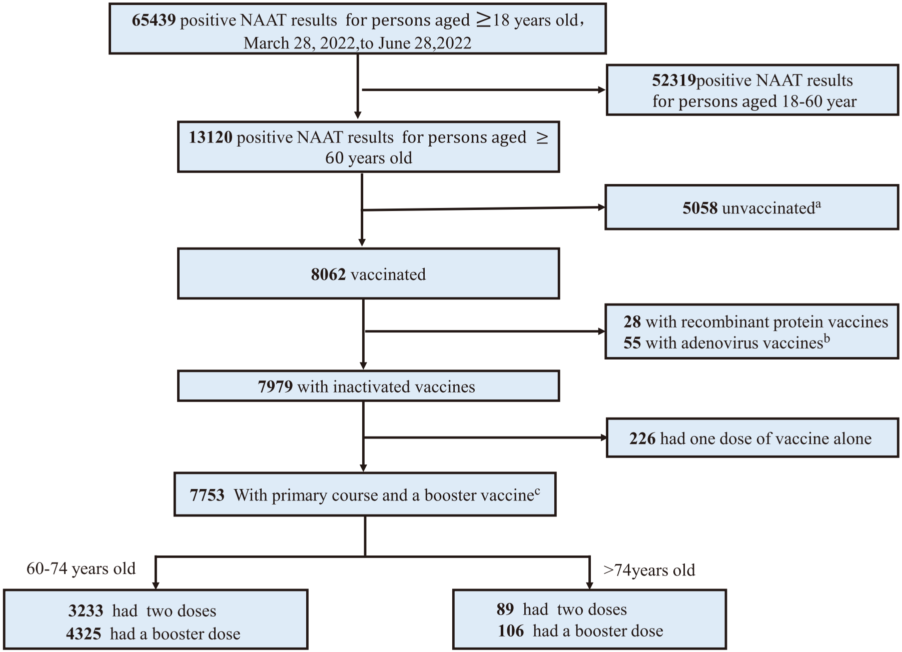
65439 positive NAAT results for persons aged ≥18 years old,
March 28, 2022,to June 28,2022
52319positive NAAT results
for persons aged 18-60 year
13120 positive NAAT results for persons aged ≥
60 years old
5058 unvaccinated[a]
8062 vaccinated
28 with recombinant protein vaccines
55 with adenovirus vaccines[b]
7979 with inactivated vaccines
226 had one dose of vaccine alone
7753 With primary course and a booster vaccine[c]
60-74 years old
>74years old
3233 had two doses
4325 had a booster dose
89 had two doses
106 had a booster dose

Figure S2.

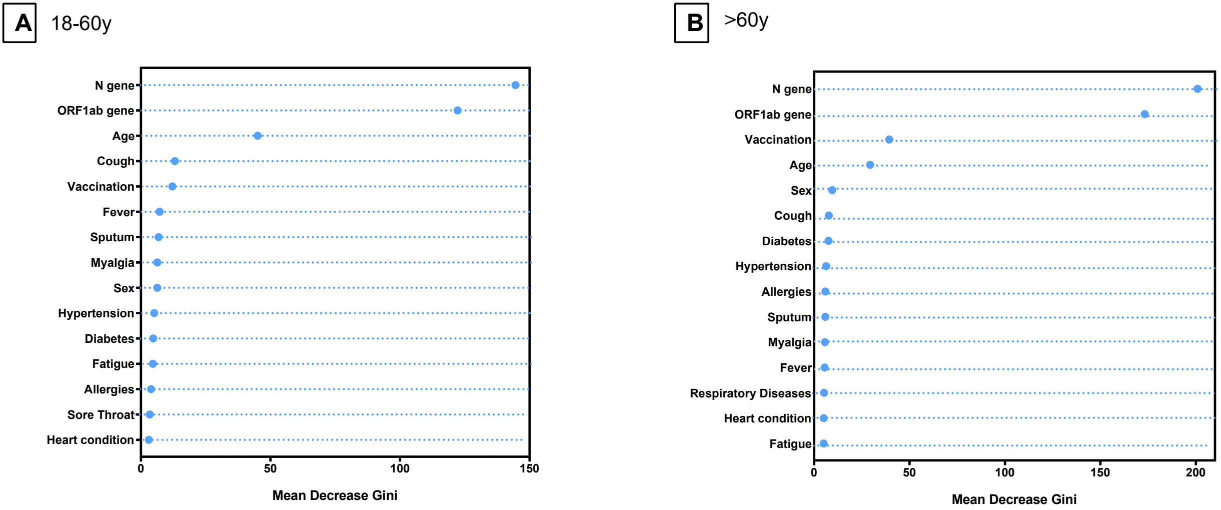
A
18-60y
N gene
ORF1ab gene
Age
Cough
Vaccination
Fever
Sputum
Myalgia
Sex
Hypertension
Diabetes
Fatigue
Allergies
Sore Throat
Heart condition
0
50
100
150
Mean Decrease Gini
B
>60y
N gene
ORF1ab gene
Vaccination
Age
Sex
Cough
Diabetes
Hypertension
Allergies
Sputum
Myalgia
Fever
Respiratory Diseases
Heart condition
Fatigue
0
50
100
150
200
Mean Decrease Gini
